# Cotranslational protein folding through non-native structural intermediates

**DOI:** 10.1126/sciadv.ady2211

**Published:** 2025-09-05

**Authors:** Siyu Wang, Amir Bitran, Ekaterina Samatova, Eugene I. Shakhnovich, Marina V. Rodnina

**Affiliations:** ^1^ Department of Physical Biochemistry, Max Planck Institute for Multidisciplinary Sciences, Göttingen 37077, Germany.; ^2^ Department of Chemistry and Chemical Biology, Harvard University, 12 Oxford Street, Cambridge, MA 02138, USA.

## Abstract

Cotranslational protein folding follows a distinct pathway shaped by the vectorial emergence of the peptide and spatial constraints of the ribosome exit tunnel. Variations in translation rhythm can cause misfolding linked to disease; however, predicting cotranslational folding pathways remains challenging. Here, we computationally predict and experimentally validate a vectorial hierarchy of folding resolved at the atomistic level, where early intermediates are stabilized through non-native hydrophobic interactions before rearranging into the native-like fold. Disrupting these interactions destabilizes intermediates and impairs folding. The chaperone trigger factor alters the cotranslational folding pathway by keeping the nascent peptide dynamic until the full domain emerges. Our results highlight an unexpected role of surface-exposed residues in protein folding on the ribosome and provide tools to improve folding prediction and protein design.

## Teaser

Early folding intermediates form via non-native contacts and are reshaped by chaperones during protein synthesis on the ribosome.

## INTRODUCTION

**Fig. 1.**
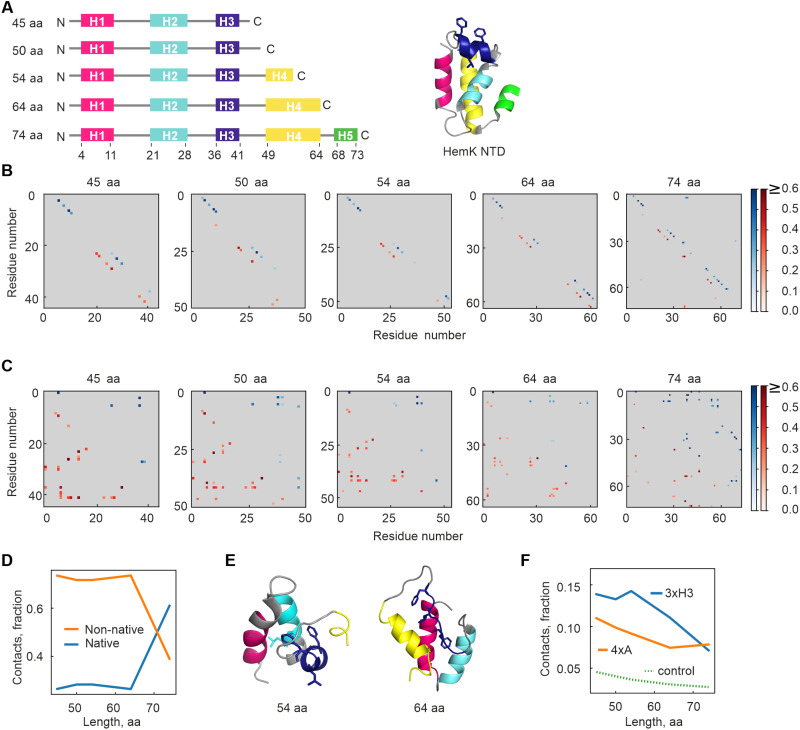
Simulations predict an early non-native folding intermediate of HemK NTD stabilized by hydrophobic interactions. (**A**) Schematic representation of simulated constructs, with helices labeled H1 to H5. Full-length HemK NTD (amino acids 1 to 73) forms a stable, autonomously folding domain of five α helices. (**B**) Average contact maps at various chain lengths for α-carbon interactions. Blue dots (top-right diagonal) represent native helical contacts between residues *i* and *i* + 3, while red dots (bottom-left diagonal) indicate slightly register-shifted, non-native helical contacts. Native contacts (blue, top diagonals) and non-native contacts (red, bottom diagonals) are scaled by probability (color bar) with contacts greater than 0.2 shown. (**C**) Same as (B) but for side-chain contacts (≤5-Å distance between side-chain centers) at different protein lengths. Side-chain interactions typically span longer sequence distances than α-carbon interactions, resulting in contact matrix elements far from the diagonal, a feature seldom observed in (B). (**D**) Fraction of native versus non-native side-chain contacts at each protein length. (**E**) Sample simulation snapshots of non-native intermediates at 54– and 64–amino acid chain length. 3xH3 residues (F^38^, L^40^, or F^42^) are shown in as dark blue sticks. (**F**) Fraction of side-chain contacts at each length involving 3xH3 (blue) or 4xA (L^27^, L^28^, L^55^, or L^58^, orange). Values are normalized by the number of residues in each group. For control, residue numbers were randomly shuffled, and 3xH3 contact frequencies recalculated. aa, amino acids.

In all cells, proteins begin to fold into their functional structures during synthesis on the ribosome (*1*–*4*). Unlike folding in solution, where the full sequence is available, cotranslational folding occurs vectorially as the nascent protein emerges from the ribosome exit tunnel, allowing early folding before synthesis is complete (*5*–*12*). The ribosome orchestrates this process by imposing spatial constraints, interacting with the nascent peptide, and modulating the structural dynamics of the emerging domains, which collectively shape the folding pathway. Interactions with ribosomal components, including ribosomal RNA and proteins lining the exit tunnel, influence the folding trajectory by stabilizing specific conformations and regulating structural transitions (*6*, *12*–*20*). Upon emerging from the ribosome and release into the cytosol, some proteins can undergo further folding/refolding cycles with or without the help of molecular chaperones, while others are not refoldable (*21*–*24*). Disruptions of cotranslational folding caused by variations in local translation rates or mutations can lead to misfolding and disease (*25*–*31*), highlighting the importance of this process for protein homeostasis. While many proteins start folding inside the ribosome exit tunnel, the guiding principles of cotranslational folding remain unclear, as intermediates are transient, and ribosomal interactions complicate structural determination. Here, we combine computational and experimental approaches to reconstruct the cotranslational folding pathway using the N-terminal domain (NTD) of HemK as a model protein. HemK NTD is a five-helix bundle (Fig. 1A) that folds rapidly in a two-state concerted transition in solution, whereas, on the ribosome, folding is sequential, involving α-helix formation and stepwise compaction (*9*, *20*, *32*). We gain insights into atomistic structure and dynamics of cotranslational intermediates, revealing crucial non-native interactions, characterize their formation and conversion into final native structure, and demonstrate the role of the chaperone trigger factor (TF) in resolving non-native interaction during cotranslational folding.

## RESULTS

### Computational predictions of folding hierarchy

To identify key residues guiding this process, we performed atomistic simulations using MCPU (Monte Carlo Protein Unfolding)/DBFOLD, a Monte Carlo (MC) simulation pipeline that predicts protein folding pathways while accounting for non-native interactions (*33*). We first validate that the wild-type (WT) full-length HemK NTD is well folded at low temperatures and undergoes a cooperative two-state thermal denaturation in our simulation, consistent with experiments (fig. S1A) (*9*). Although the computational platform does not explicitly model the ribosome, these simulations recapitulate intrinsic properties of cotranslational folding, such as the sequential emergence of the peptide and its propensity to form secondary and tertiary structures. They also provide insights into the thermodynamic stability and dimensions of folding intermediates, which determine whether structural elements can form inside the narrow ribosome exit tunnel. To mimic the vectorial nature of cotranslational folding, we ran equilibrium simulations of the domain truncated at various C-terminal positions (Fig. 1A). At intermediate lengths (45 to 64 amino acids), the protein readily forms native-like α helices (Fig. 1B) but adopts persistent non-native tertiary structures (~70% non-native contacts; Fig. 1, B and C), which are favored over native-like structures by 2 to 3 *k*_B_*T* (fig. S1, B and C). This energy difference suggests that, early during synthesis, non-native states are about 7 to 20 times more likely than native-like ones. Native contacts become more important only at full-length (74 amino acids) (Fig. 1D), with a stability gain of ~4 *k*_B_*T* (~55-fold) relative to the unfolded state (fig. S1C).

Next, we analyzed molecular interactions stabilizing these intermediates. Three hydrophobic residues in helix 3 (Phe^38^, Leu^40^, and Phe^42^, hereafter denoted as 3xH3), which are typically solvent-exposed in the native state, are frequently buried in non-native intermediates (Fig. 1E). At short chain lengths, 3xH3 residues account for ~15% of side-chain contacts, exceeding random expectations (Fig. 1F). In contrast, native hydrophobic core residues [Leu^27^, Leu^28^, Leu^55^, and Leu^58^, previously denoted as 4xA; (*9*)] account for only ~10% of interactions at shorter lengths but become dominant at later stages. At full length, 4xA residues dominate, stabilizing the native state, although non-native 3xH3 contacts are still visible, likely due to structural fluctuations that transiently bury these hydrophobics within the core, despite their solvent-exposed nature in the crystal structure. To assess their role, we reran simulations with mutants in which either 3xH3 or 4xA residues were replaced with Ala. Unexpectedly, at full length, both mutations destabilized the native state, despite 3xH3 residues being expected to become surface-exposed, although the destabilizing effect was more pronounced for the hydrophobic core mutation, 4xA (fig. S1, C to E), consistent with the observed hierarchy of contact formation (Fig. 1F). Moreover, 3xH3, but not the 4xA, affected the stability of folding intermediates before completion of synthesis (fig. S1, B to D). These results suggest a vectorial hierarchy in cotranslational intermediates, with 3xH3 residues initially forming a non-native core that rearranges toward native-like, albeit destabilized, domain as translation progresses.

### Non-native interactions in cotranslational folding

**Fig. 2.**
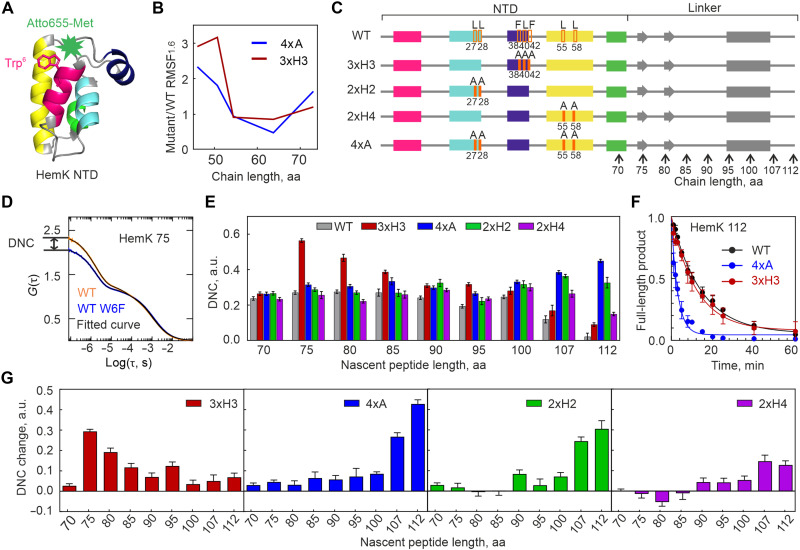
HemK NTD forms non-native intermediates during translation. (**A**) Structure of HemK NTD (Protein Data Bank 1T43) with the N-terminal fluorophore highlighted in green and Trp^6^ shown in magenta (stick representation). (**B**) Predicted RMSF in the distance between residues 1 and 6 for 3xH3 and 4xA mutants relative to WT at equivalent nascent chain lengths. (**C**) Schematic of constructs for PET experiments, including the NTD (amino acids 1 to 72) and the linker (gray, amino acids 73 to 112) connecting the emerging domain to the peptidyl transferase center of the ribosome. As linker, native HemK sequence was used, which entails potential secondary structure elements such as two short β strands and an α helix as indicated. Vertical arrows indicate the amino acid length of nascent-chain constructs used in the experiments. Orange rectangles indicate mutation sites. (**D**) Example of PET-FCS data with fitted curves (black). Dynamicity of nascent chain (DNC), a qualitative parameter used to assess the fraction of molecules undergoing conformational fluctuations, was determined from the *G*(0) difference between W6 and W6F ribosome-nascent chain constructs. (**E**) DNC values for all tested constructs determined from the *G*(0) differences as described in (D); error bars represent SEM calculated from at least eight biological replicates (*N* ≥ 8). (**F**) Protease digestion of HemK112 nascent chains. Full-length peptide intensity at time zero was used for normalization. Data represent mean values with SD of three biological replicates (*N* = 3). Smooth lines represent single-exponential fits used to calculate the decay constant (τ). τ = 14.4 ± 3.7 min (WT), 12.0 ± 1.2 min (3xH3), and 3.0 ± 0.3 min (4xA) (means ± SD). (**G**) DNC changes for the 3xH3, 4xA, 2xH2, and 2xH4 mutants relative to the WT. Error bars are SEM calculated from at least eight biological replicates (*N* ≥ 8). aa, amino acids. a.u., arbitrary units.

We then experimentally tested the simulation predictions using fluorescence correlation spectroscopy (FCS) combined with fluorescence quenching via photoinduced electron transfer (PET). We used a fluorescence label (Atto655) at Met^1^, which is quenched upon contacting Trp^6^ in the nascent chain and becomes unquenched as it moves away, reflecting protein structural dynamics in the microsecond to millisecond time range (Fig. 2A) (*34*, *35*). To test its sensitivity to hierarchical folding, we calculated the root mean square fluctuation (RMSF) between residues 1 and 6 for the WT and 3xH3 and 4xA mutants. Simulations predict that 3xH3 mutations increase dynamics when the nascent peptide is short, while this effect diminishes at longer chain lengths. In contrast, 4xA mutations have a stronger effect on dynamics at full length than 3xH3 mutation (Fig. 2B), consistent with their role in native folding (*9*). If folding on the ribosome follows the same pathway as in the computer simulation, we expect similar tendencies to appear during translation, although the magnitude of the effect may differ. This is because the ribosome is known to destabilize nascent protein structures, with the extent of destabilization varying depending on whether the domain is still inside the tunnel or has been extruded (*20*).

To measure the effects of mutations, we prepared ribosome-nascent chain complexes (RNCs) with WT, 3xH3, and 4xA constructs (Fig. 2C and fig. S2; Materials and Methods). Additionally, we designed double Ala mutants in helix H2 (Leu^27^Ala and Leu^28^Ala, denoted as 2xH2) and helix H4 (Leu^55^Ala and Leu^58^Ala, denoted as 2xH4) to assess individual contributions to folding. Because compact tertiary structures comparable to those observed in computer simulations form only after a portion of the peptide exits the ribosome, we tested constructs of different lengths, starting from 70 amino acids, which is completely buried inside the tunnel, up to 112 amino acids, which is known to expose the whole HemK NTD outside the tunnel (Fig. 2C) (*9*). To estimate the contribution of the ribosome-induced quenching effects, we prepared control constructs (W6F) where Trp^6^ was replaced by a nonquenching Phe. PET-FCS autocorrelation functions [ACFs; denoted as *G*(τ ) in Fig. 2D] showed a millisecond diffusion time identical for all RNCs (*20*) and a biphasic PET signal spanning the microsecond timescale. The W6F signal monitors interactions on the nascent chain N terminus with quenchers on the ribosome, such as Trp or guanine residues, whereas the signal for W6 is a sum of the inter- and intramolecular quenching (fig. S3, A and B). The *Y*-intercepts [*G*(0)] of ACF curves, representing quenching event frequency (*36*), enabled a simple estimation of the dynamicity of nascent chain (DNC) values from the difference between W6 and W6F ACFs (Fig. 2E; Materials and Methods). Higher DNC values indicate dynamic nascent chains, while stably folded molecules have values close to zero (*20*).

The WT nascent chains remain dynamic from 70 to 105 amino acids, gradually compacting beyond 107 amino acids as the NTD emerges from the exit tunnel and folds, in agreement with previous findings (Fig. 2, E and F, and fig. S3C) (*20*). The 3xH3 mutant exhibits WT-like dynamics at 70 amino acids but becomes substantially more dynamic at 75 to 100 amino acids, indicating destabilization of intermediates. At longer lengths, both WT and 3xH3 reach compact states, although 3xH3 remains somewhat more dynamic [Fig. 2 (E and G), consistent with the prediction of fig. S1 (C and D)]. Mutations in 4xA residues have distinctly different effects. The 2xH2, 2xH4, and 4xA mutations have little impact on early folding, with only mild destabilization between 90 and 100 amino acids. This supports the idea that hydrophobic core residues do not contribute to early folding intermediates (*20*). However, destabilization becomes significant beyond 107 amino acids, coinciding with the emergence of the domain from the ribosome tunnel. The effect is most pronounced for the mutations in helix 2, likely disrupting early H1-H2 interactions (*32*). Notably, comparison of the W6F variants shows that the 3xH3 and 4xA mutations do not significantly alter intermolecular PET quenching, indicating that their interactions with the ribosome remain largely unchanged (fig. S3D).

Limited proteolysis assays, which use proteases to selectively cleave accessible, flexible regions of a protein, can reveal differences in folding and structural stability. In these assays, the full-length WT and 3xH3 mutant are degraded slowly, consistent with a compact conformation that limits protease access, whereas the 4xA is rapidly degraded, indicating a destabilized and more accessible structure (Fig. 2F and fig. S3C). These findings indicate that 3xH3 residues stabilize early non-native intermediates, while 4xA residues are crucial for native state folding of the full-length domain, in agreement with computational predictions.

### Impact of non-native interactions on nascent chain compaction

**Fig. 3.**
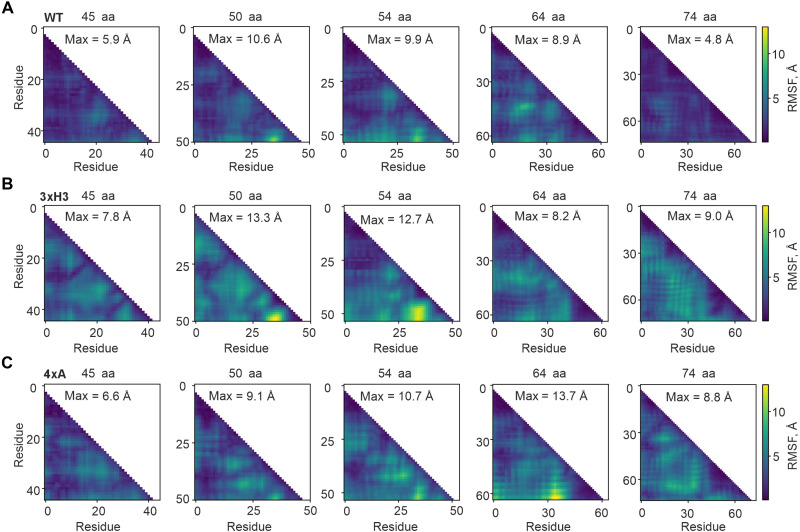
Disrupting non-native interactions destabilizes full-length domain folding. Heatmaps show RMSF, defined as the RMSF in the distances between residue pairs relative to their average distances, at different nascent chain lengths. RMSF values are color coded according to the color bars to the right of each row. The maximum RMSF value in each heatmap is indicated. (**A**) WT HemK NTD. (**B**) 3xH3. (**C**) 4xA. aa, amino acids.

On the ribosome, 3xH3 mutation disrupts folding into a non-native intermediate when the nascent chain reaches ~75 amino acids. This non-native intermediate includes a ~50 amino acids compact folding unit and a ~25–amino acid linker (*9*, *32*), comprising an intermediate with helix 3 docking onto a unit formed by native contacts between helices 1 and 2 (Fig. 1), which coincides with a major force-generating event during cotranslational folding of HemK NTD (*20*). To characterize this intermediate, we analyzed domain-wide dynamics at the atomic level by calculating the RMSF of pairwise distances between all amino acids relative to their average distance (Fig. 3). Although the structures of HemK (full-length or intermediates) are much more stable in solution than on the ribosome (*20*), such comparisons provide insights into the relative effects of mutations. In WT HemK NTD, RMSF values at intermediate peptide lengths largely match those of the full-length protein, with only a few localized fluctuations. In contrast, the 3xH3 mutant at lengths corresponding to the ~50–amino acid folding unit (45 to 55 amino acids) shows significantly higher RMSF (Fig. 3, A and B), particularly in the linker between helices 2 and 3 (residues 32 to 37) and at the C terminus, suggesting that non-native interactions play a role in stabilizing folding intermediates as they emerge from the ribosome.

These findings are further supported by calculations of the radius of gyration (*R*_g_) distribution for each length (fig. S4), representing the degree of domain compactness. WT HemK NTD remains consistently compact, with a mean *R*_g_ at 45 to 50 amino acids differing by no more than 1 Å from the native structure and exhibiting a narrow *R*_g_ distribution (<2 Å). This is consistent with the predicted WT *R*_g_ of about 10 Å, a structural unit size compatible with the known size of the ribosomal tunnel vestibule, and indicates that WT intermediates are compact despite the non-native interactions that stabilize them. The 3xH3 mutant, however, adopts more expanded states with a broad *R*_g_ distribution at 45 to 50 amino acids, reinforcing the idea that non-native interactions help form a compact intermediate suitable for helix 3 docking inside the exit tunnel. In contrast, the 4xA mutation is similar to the WT in RMSF (Fig. 3C) and *R*_g_ distribution (fig. S4, A to D) at short nascent peptide lengths but becomes different for the WT at 64 and 74 amino acids, where the protein transitions into a native-like state stabilized by 4xA residues.

### Real-time analysis of folding

**Fig. 4.**
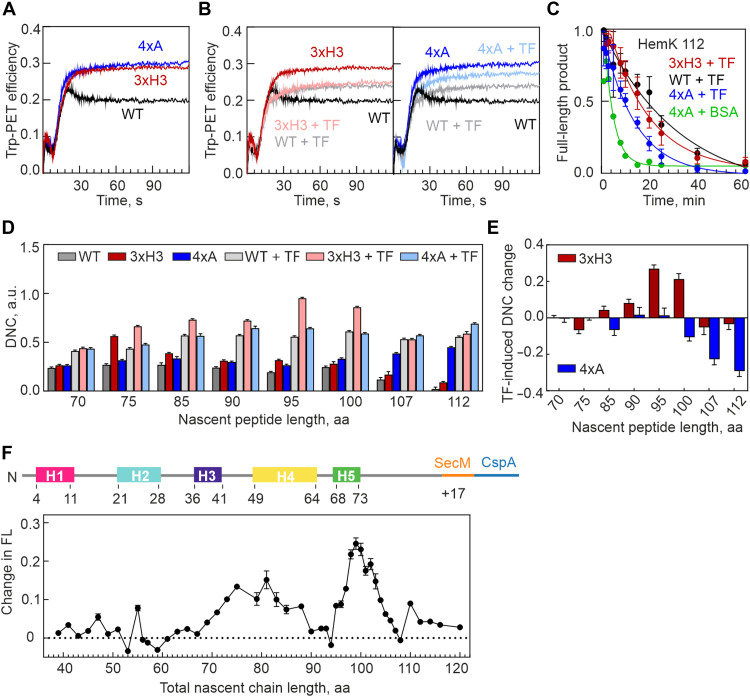
TF alleviates misfolding by destabilizing intermediates and the final domain fold. (**A**). Stopped-flow time course of HemK NTD cotranslational compaction monitored by PET between Atto655-Met^1^ and Trp^6^ in the nascent chain. Time zero corresponds to translation start upon mixing the 70*S*–mRNA–Atto655–Met-tRNA^fMet^ complex with EF-Tu, EF-G and aminoacyl-tRNAs. (**B**) TF effect on cotranslational folding of WT, 3xH3 (left), and 4xA (right). Note that the traces for WT + TF (light gray) and 3xH3 (light red) are almost identical. (**C**) Time courses of protease digestion in the presence of TF. Data represent means ± SD of three biological replicates (*N* = 3). τ = 31.7 ± 6.0 min (WT + TF), 22.4 ± 3.1 min (3xH3 + TF), 13.5 ± 1.5 min (4xA + TF), and 3.7 ± 0.7 min (4xA + BSA). (**D**) TF effect on DNC monitored by PET-FSC as in Fig. 2. Error bars represent SEM calculated from at least eight biological replicates (*N* ≥ 8). (**E**) Relative TF-induced DNC change for 3xH3 and 4xA compared to the WT + TF nascent chain of the same length. Plotted is the dynamicity change upon TF addition of the mutant versus WT. Error bars represent SEM calculated from at least eight biological replicates (*N* ≥ 8). (**F**) TF-induced change in the FPA profile of WT nascent peptide. The total nascent chain length includes the HemK nascent peptide and the SecM stalling sequence. Data represent means ± SD of three biological replicates (*N* = 3). aa, amino acids.

Although the mutated residues in the 3xH3 mutant are largely solvent-exposed in the native structure, the full-length 3xH3 protein appears more dynamic than the WT in both computer simulations (Fig. 3B and figs. S1D and S4E) and PET-FCS measurements (Fig. 2G). Furthermore, simulations indicate that the full-length 3xH3 mutant exists as a dynamic ensemble of conformations, including favorable non-native interactions (Fig. 3B and figs. S1 and S4). On the other hand, protease protection experiments suggest a compact fold comparable to the WT (Fig. 2F and fig. S3C). This apparent contradiction prompted us to investigate the folding pathway using an orthogonal approach. While FCS captures millisecond-scale dynamics and protease protection assays reflect minute-scale stability, we bridged these timescales by measuring real-time PET efficiency changes using a stopped-flow apparatus. We start translation by rapid mixing of translation elongation factors and aminoacyl-tRNAs with the ribosome in complex with an mRNA and BodipyFL-Met-tRNA^fMet^ and monitor changes in Bodipy fluorescence as the nascent peptide is synthesized. As for PET-FSC experiments, we perform these experiments with W6 and W6F peptides to distinguish between the contributions of the ribosome and nascent chain folding (Materials and Methods) (*20*). During WT HemK NTD synthesis, PET efficiency initially increases, suggesting the formation of an intermediate where the fluorescent reporter is near Trp^6^, and then decreases (Fig. 4A). The decrease coincides with full-length protein synthesis (fig. S5, A and B), suggesting the transition to a native-like structure (*20*). As expected, the 4xA mutant does not undergo this final rearrangement (Fig. 4A). The full length 3xH3 mutant is also trapped in a non-native (Fig. 4A) yet compact (Fig. 2F) conformation, consistent with simulation results (Fig. 3B and figs. S1 and S4). Notably, PET efficiency changes due to intermolecular interactions with the ribosome monitored by the W6F variants were identical for WT, 3xH3, and 4xA constructs (fig. S5C). These results together with computer simulations (Fig. 3) demonstrate that disrupting non-native hydrophobic interactions that guide hierarchical folding can lead to a compact but partially misfolded protein.

### Role of TF

In cells, molecular chaperones prevent protein misfolding. This prompted us to ask whether chaperones can also correct cotranslational misfolding caused by the alterations in folding pathway. TF, a cotranslational chaperone that binds at the ribosome tunnel exit, interacts directly with nascent peptides (*37*). In real-time translation experiments, TF prevents relaxation of the WT HemK domain (Fig. 4B), with effects visible after 10 to 14 s (fig. S5D) corresponding to the synthesis of the first 70 to 90 amino acids (fig. S5B). This suggests that TF engages early, influencing folding by binding to a crucial intermediate that forms during the synthesis. When TF is added during 3xH3 mutant translation, PET efficiency becomes identical to that of the WT (Fig. 4B), eliminating the folding differences between the WT and 3xH3 variant. Notably, the overall domain structure remains compact (Fig. 4C). These data indicate that, by binding to an early intermediate, TF does not block the overall domain compaction but delays final folding, which might avoid formation of the compact misfolded forms arising when the 3xH3 interactions cannot form. In contrast, while TF affects structure of the 4xA mutant (Fig. 4, B and C), its final PET efficiency in 4xA remains higher than in WT, suggesting that TF cannot eliminate the folding defect that arises when the hydrophobic core is disrupted.

To examine how TF affects protein fluctuations on a microsecond timescale, we performed PET-FCS experiments. TF increases dynamicity in both WT and mutant proteins at all peptide lengths (Fig. 4D and fig. S5, E and F), supporting the idea that TF destabilizes nascent peptide folding on the ribosome. However, the magnitude of the effect varies with the nascent chain: TF increases fluctuations in 3xH3 more than in the WT at 95 to 100 amino acids (Fig. 4E). For 4xA, the effect is similar to that of the WT up to a chain length of 100 amino acids. However, at longer chain lengths, the effect is reduced compared to WT, possibly due to stabilization by bound TF of a domain with a defective hydrophobic core (Fig. 4C and fig. S5G).

As stopped-flow kinetics indicated early TF recruitment to the nascent chain, while PET-FCS suggested that its main effect occurs at longer chain lengths, we examined TF’s influence on folding using an orthogonal approach. We performed high-resolution force profile analysis (FPA) (*5*) using constructs encoding HemK NTD peptides of varying lengths (8 to 103 amino acids), each followed by 17 codons of the SecM arrest peptide and a 20–amino acid reporter (*20*). In the absence of tension, translation stalls at the SecM arrest motif. When mechanical force is generated, translational arrest is relieved, allowing synthesis to continue. Without TF, the FPA profile reveals distinct peaks corresponding to the sequential folding of the NTD through stepwise helix formation and docking (figs. S5H and S6) (*20*). TF increases tension, particularly at nascent chain lengths of 72 to 87 amino acids and 95 to 103 amino acids (Fig. 4F). Once the peptide reaches 112 amino acids, both profiles converge at a baseline level (fig. S5H), indicating that the emerged NTD does not exert tension on the ribosome regardless of TF’s presence. The increased tension at 72 to 87 amino acids coincides with TF’s early recruitment observed in stopped-flow experiments, while the peak at 95 to 103 amino acids overlaps with the length at which the maximum destabilizing effect of TF on folding is observed. This additional tension reflects mechanical force preventing the final compaction of the full-length nascent chain as it emerges from the ribosome.

## DISCUSSION

Our simulations predict cotranslational folding intermediates at atomistic level of detail. The key 3xH3 residues were identified de novo through these simulations, as they were not previously known to play a role in folding, underscoring the predictive power of the approach. Experimental data confirm the presence of these intermediates, revealing that the cotranslational folding of HemK NTD involves well-defined structures stabilized by specific non-native contacts as the nascent peptide emerges from the ribosome. The temporal hierarchy of these intermediates defines the folding pathway, guiding the nascent chain toward its final native conformation. These non-native intermediates may help compact the nascent chain within the narrow ribosomal tunnel, facilitating proper folding. Our *R*_g_ analysis suggests that, without these interactions, nascent chains would be less compact, which could hinder folding and increase the risk of misfolding. Mutations that disrupt critical non-native interactions not only alter the folding pathway but also result in a different ensemble of conformations at full length. While the equilibrium in this ensemble is shifted toward a compact state, as shown by protease protection experiments, the local interactions remain dynamic, suggesting local misfolding. TF engages with nascent chains early during translation, at a stage when hydrophobic interactions begin to form a non-native compact intermediate and modulate the nascent chain dynamics of the growing peptide. TF destabilizes both native-like and potentially misfolded structures, thereby delaying final folding (*38*). The mechanical force generated by interactions between TF, the nascent peptide, and the ribosome destabilizes prematurely folded structures, ensuring that the peptide remains flexible and capable of adopting its correct native conformation once the complete domain sequence emerges from the ribosome and the nascent chained is released from the ribosome and TF. We show that TF can mitigate cotranslational misfolding even in proteins that are not obligate TF clients, such as HemK, by delaying their final compaction into a stable fold. Once the nascent chain is released from both the ribosome and TF, these proteins have a chance to attain their stable correct structure. Misfolding due to altered folding pathways may become critical under stress conditions, when chaperone capacity is overwhelmed. This is particularly important for proteins that are inherently unable to refold in solution (*22*), as cotranslational TF-mediated control of their final packing may represent the last opportunity for refolding before potentially misfolded protein is released into the cytoplasm.

While the native fold is encoded in the sequence, evolutionary adaptation of cotranslational folding likely involves residues that do not contribute to the final structure but are essential for guiding the formation of folding intermediates. This reflects the vectorial nature of protein synthesis and the interactions of the nascent chains with the ribosome exit tunnel. Replacing such seemingly neutral surface residues can disrupt intermediates and lead to misfolding. This insight is crucial for understanding how the rhythm of translation influences protein folding, and it has practical implications for the design of novel protein sequences. For example, it may help explain why variations in translation rates at codons encoding amino acids that do not directly participate in folding can cause disease and why mutations of surface-exposed residues that appear nonessential in the native structure can still be detrimental to proper folding.

Our results also demonstrate that simulations of vectorially truncated N-terminal peptides folding in solution can predict folding intermediates as peptides exit the ribosome. Although direct structural validation of these simulated intermediates remains challenging, the observed agreement between predicted trends and experimental effects of key interacting residues supports that the simulations capture relevant, physiologically meaningful intermediate states, underscoring the predictive power of our computational approach. To date, this computational approach has been applied to map free energy landscapes and identify cotranslational folding intermediates in proteins whose lengths varied from 100 amino acids to more than 300 amino acids in length (*39*–*42*). In future applications, simulations could incorporate TF-like effects, such as promoting dynamics or delaying premature compaction, to predict which mutations might be recoverable by TF and which are irreversibly misfolding-prone. This would open the door to more predictive, mutation-specific modeling of chaperone-assisted cotranslational folding.

Furthermore, the ability to predict residues essential for early folding opens the door to improved experimental design. For example, simulations could help identify labeling sites to monitor folding transitions while avoiding disruption of critical structural elements. This could refine residue selection for fluorescence resonance energy transfer, nuclear magnetic resonance, or proteolysis-based approaches to studying cotranslational folding in vitro and in vivo. Overall, this strategy provides a framework for constructing cotranslational folding profiles for diverse proteins, including those prone to misfolding. While further method development is needed to simulate interactions with chaperones, biogenesis factors, or assembly partners in detail, the current findings establish a foundation for such work and highlight the predictive value of folding simulations during translation.

## MATERIALS AND METHODS

### Computational methods

#### All-atom MC simulations

Simulations of all HemK constructs were carried out using the MCPU, an all-atom MC simulation algorithm that uses a knowledge-based potential function to compute interaction energies between all residue pairs while accounting for both native and non-native interactions, as described previously (*33*). We prepared starting structures for simulations from the Protein Data Bank file 1T43, with Arg at position 34 (Arg^34^) replaced with Lys to match the construct used in experiments. Residues 75 onward were deleted to produce a 74–amino acid HemK NTD fragment containing all five helices; this construct is hereafter referred to as full-length HemK NTD. We then generated additional truncations from the C terminus to produce variable-length N-terminal fragments as described in the text. This process was repeated to generate full-length and truncated HemK variants with 3xH3 (F^38^, L^40^, or F^42^) and 4xA (L^27^, L^28^, L^55^, or L^58^) replacements with Ala. The sequences for the respective constructs were used to generate sequence-specific parameters for local conformational energy calculations as described (*43*). Each starting structure was equilibrated in the MCPU potential function using the low-temperature replica-exchange equilibration protocol (*33*, *39*) with a distance cutoff of 8.5 Å used to compute α-carbon contacts for umbrella biasing. The purpose of umbrella biasing is to speed up simulation convergence by encouraging a system to cross high energy barriers. This is achieved by adding a harmonic biasing potential along a desired order parameter—in our case, native contacts—to promote exploration of otherwise infrequently visited states. This biasing term is subsequently accounted for to calculate unbiased average thermodynamic quantities (*33*). Before production runs, a low simulation temperature of 0.1 (in units of simulation energy) was used to relax the structure, and, following 20 million MC steps of equilibration, the lowest energy structure among all replicas was identified and used as the starting structure for subsequent production simulations of the respective construct.

Production runs were then carried out for 200 million MC steps at a range of temperatures from *T* = 0.4 to *T* = 1.0 in increments of 0.025, and umbrella biasing set points ranging from 0 to the number of α-carbon contacts in the equilibrated structure, in increments of 10 contacts, producing a grid of simulations in set point and temperature space. Replica exchanges were periodically attempted between neighboring simulations as in (*33*) to help improve convergence. Following completion of the 200 million MC step simulation, the second 100 million steps were used to calculate equilibrium properties. This was justified by the observation that the sliding-window averaged energy as a function of simulation step generally ceased changing appreciably after 100 million MC steps, indicating convergence.

#### Computation of thermodynamic properties from all-atom simulations

To analyze converged simulations for each construct, we began by computing the average number of native contacts, defined as contacts that present in the equilibrated native structure, as a function of simulation temperature using MBAR (*44*), a statistically optimal approach for estimating thermodynamic properties from biased simulations. This method can be used to calculate thermal melting curves like those shown in fig. S1A while correcting for biases introduced during umbrella sampling. From this melting curve, we identified the melting temperature (T_M_) for WT full-length NTD as *T*_M_ = 0.525, the temperature at which the average number of native contacts is closest to the half-maximal value. We then constructed more detailed folding landscapes using the substructure approach detailed in (*33*).

In this method, clusters of cooperatively forming native contacts are identified from the contact map; in the case of HemK, we used an 8.5-Å distance cutoff for contacts excluding residues less than eight apart in primary sequence, and we clustered together contacts separated by no more than four Manhattan distance units on the contact map, provided that the resulting clusters were at least seven residues in size. The resulting clusters, known as substructures are labeled A to E in fig. S1E. Each simulation snapshot was then assigned to a topological configuration, which is a label that identifies the substructures that are formed or broken in that particular snapshot. We then computed a potential of mean force as a function of topological configuration using MBAR as in fig. S1 (C to E).

As an alternative approach to characterize native versus non-native folding propensities, we generated average contact maps at a fixed temperature of *T* = 0.45 (or *T* = 0.86 *T*_M_ with *T*_M_ = 0.525) as shown in Fig. 1. Two types of contact maps were generated. In the first, we computed centroid positions for each side chain in every snapshot. We then generated a side-chain contact map for each snapshot by identifying all pairs of side chains for which the distance between the respective centroids is less than 5 Å, provided they are at least four residues apart in primary sequence. We then computed a thermally averaged contact map using MBAR. Alternatively, the same calculation was performed using α-carbons with the same distance cutoff. From side-chain contact maps, we identified native contacts as those within one Manhattan distance unit of contacts present in the equilibrated native structure; this distance tolerance allows slightly register-shifted contacts to still be counted as native-like. This allows us to calculate the fraction of contacts in each snapshot that are native or non-native. We also used these contact maps to count the total number of contacts involving 3xH3 or 4xA residues, and these values were normalized by the number of residues in each group. As a control, this calculation was repeated for 3xH3 residues in contact maps where residue indices were randomly reshuffled. All these quantities were thermally averaged across snapshots using the MBAR algorithm.

For each snapshot from our converged simulation at temperature *T* = 0.45, we computed the radius of gyration as follows

$$R_{g}=\sqrt{\frac{1}{N}\sum_{i=1}^{N}{\left(r_{i}-<r>\right)}^{2}}$$

where *i* indicates residues, *N* is the total number of residues in the construct, $r_{i}$ is the position of the *i*th α-carbon, and <*r*> is the average position of all α-carbons in the structure. This quantity was computed separately for each snapshot, and, then, MBAR was used to obtain a probability distribution *P*($R_{g}$ ) and average <$R_{g}$> over snapshots. These quantities are shown in fig. S4. As a control, we calculated the average *R*_g_ that would be expected for a perfectly disordered random coil as

$$<R_{g}>=\frac{1}{\sqrt{6}}3.6N^{0.588}$$

where 3.6 is the approximate contour length of an individual amino acid (in angstroms), *N* is the number of residues, and 0.588 is the scaling exponent for the end-to-end distance of a self-avoiding random walk in three dimensions (*45*). These values are shown in fig. S4.

Last, as a metric for dynamicity of a given construct, we used MBAR to calculate, for each residue pair, the distribution of distances between the respective alpha carbons. From these distributions, we calculated the mean squared fluctuation relative to the average distance, and taking the square root of this quantity gives the RMSF. These RMSF values are shown for each residue pair in Fig. 3. The pairwise value corresponding to residues 1 and 6 was used to generate Fig. 2B.

### Biochemical methods

#### Buffers and reagents

Biochemical experiments were carried out in HiFi buffer [50 mM Hepes-HCl (pH 7.5), 70 mM NH_4_Cl, 30 mM KCl, 3.5 mM MgCl_2_, 8 mM putrescine, and 0.5 mM spermidine] or HAKM_7_ buffer [50 mM Hepes-HCl (pH 7.5), 70 mM NH_4_Cl, 30 mM KCl, and 7 mM MgCl_2_] (*46*). 70*S* ribosomes and other translational components, including initiation factors (IF1, IF2, and IF3), initiator tRNA (fMet-tRNA^fMet^), total aminoacyl-tRNA , EF-G, EF-Tu, and EF-Ts, were prepared according to published protocols (*47*, *48*).

#### Plasmid construction

The coding sequence of HemK NTD was cloned into pEX-A128 vector, which carries an ampicillin resistance cassette (Eurofins Genomics, Ebersberg, Germany). For PET-FCS measurements, the native Trp at position 6 (W6) was used, while Trp^78^ and Tyr^3^ were mutated to Phe in all WT and mutant constructs to avoid PET interactions with the N-terminal fluorescent reporter.

For the force profile assay (FPA), a series of constructs containing different length of WT HemK (amino acids 1 to 101) were designed, each followed by the 17–amino acid SecM arrest peptide (*49*), and 20–amino acid cold shock protein A (CspA) as reporter (UniProt ID: P0A9X9) was prepared (*20*). The plasmids and primers used for FPA were designed and validated according to a previously published study (*20*). RNA transcription templates for all constructs were generated using a commercial T7 RNA-polymerase forward primer (Eurofins Genomics). All constructs are listed in table S1.

#### mRNA transcription and purification

DNA templates were transcribed in vitro in a transcription buffer containing 40 mM Tris-HCl (pH 7.5), 15 mM MgCl_2_, 2 mM spermidine, 10 mM NaCl supplemented with GMP (5 mM), nucleoside triphosphates (3 mM), dithiothreitol (DTT; 10 mM), ^1^/_10_ volumes of polymerase chain reaction products, pyrophosphatase (0.005 U/μl), T7 polymerase (0.8%), and ribonuclease (RNase) inhibitor (0.2 U/μl). Transcription was carried out at 37°C for 3 hours. Following transcription, mRNA was purified using anion-exchange chromatography on a HiTrap Q HP 5 ml column (GE Healthcare). mRNA was eluted using a linear NaCl gradient (0.3 to 1.5 M NaCl) over 20 column volumes. mRNA-containing fractions were collected and precipitated by adding ^1^/_10_ volumes of 20% potassium acetate (pH 5.0) and 2.2 volumes of ethanol at −20°C. The mRNA was pelleted by centrifugation at 4000 rpm for 1 hour at 4°C and resuspended in RNase- and deoxyribonuclease-free water. The yield and integrity of the mRNA was assessed using 10% polyacrylamide gel electrophoresis with 8 M urea and the absorbance at 260 nm.

#### In vitro translation and ribosome-nascent chain complexes (RNC) purification

Translation was carried out according to established protocols (*46*) with the following modifications. 70S initiation complex (IC) was assembled by incubating 70S ribosomes (0.5 μM) with a 1.5-fold excess of IF1, IF2, and IF3 (0.75 μM each); 1.5-fold excess of BodipyFL-[^3^H]Met-tRNA^fMet^ (0.75 μM) or Atto655-[^3^H]Met-tRNA^fMet^ (0.75 μM); and 4-fold excess of mRNA (2 μM) in HAKM_7_ buffer and incubating at 37°C for 45 min. BodipyFL-[^3^H]Met-tRNA^fMet^ was used to establish efficient in vitro translation, stopped-flow experiment, and the limited proteolysis, Atto655-[^3^H] Met-tRNA^fMet^ was used for PET-FCS measurements.

The ternary complex (TC) was prepared by incubating EF-G (4 μM), EF-Ts (0.2 μM), EF-Tu (25 μM), and aminoacyl–tRNA (300 μM), DTT (2 mM), guanosine 5′-triphosphate (GTP; 2 mM), phosphoenolpyruvate (6 mM), and pyruvate kinase (0.5 mg/ml) in HAKM_7_ buffer. All components except aminoacyl–tRNA were preincubated at 37°C for 15 min, followed by addition of aminoacyl–tRNA and incubation at 37°C for 1 min to form the TC. Translation reactions were performed in HiFi buffer by combining TC (300 μM) and IC (0.04 μM) and incubating at 37°C for 5 min. For truncated mRNAs lacking the stop codon, the translated nascent chains remained bound to ribosomes, forming RNCs.

Translation efficiency was analyzed using Tris-Tricine SDS–polyacrylamide gel electrophoresis (PAGE). Translation reactions containing BodipyFL–labeled nascent chains were quenched by adding ^1^/_5_ volume of 2 M NaOH, followed by incubation at 37°C for 30 min to release the nascent peptides. The reaction was neutralized by adding an equal volume of 2 M Hepes. For Atto655-containing translation products, ^1^/_10_ volume of hydroxylamine (1.5 M) was added instead of NaOH, followed by incubation at 37°C for 1 hour. SDS sample buffer [50 mM tris-HCl (pH 6.8), 4% w/v SDS, 2% v/v 2-mercaptoethanol, and 12% w/v glycerol] was added at a 1:1 ratio. Samples were heated at 70°C for 10 min, and peptide products were separated on Tris-Tricine gel. Fluorescent peptides were visualized using an Amersham Typhoon RGB fluorescence scanner (Cytiva) with excitation wavelengths of 488 nm for Bodipy FL and 680 nm for ATTO-655.

For PET-FCS experiments, RNCs were purified by ultracentrifugation through a 1.1 M sucrose cushion in HiFi buffer using a TLA-100 rotor (Beckman Coulter) at 68,000 rpm for 1 hour at 4°C. The RNC pellet was resuspended in HiFi buffer, and RNC concentration was determined by liquid scintillation counting of the ^3^H-label using a PerkinElmer Tri-Carb 3110 TR Scintillation Liquid Analyzer. Purified RNCs were flash frozen in liquid nitrogen and stored at −80°C.

#### Photoinduced electron transfer fluorescence correlation spectroscopy

PET-FCS measurements were performed on the MicroTime 200 system (PicoQuant) built on a modified Olympus IX 73 confocal microscope equipped with 60× water immersion objective Olympus UPlanSApo 1.2 numerical aperture (Olympus UPlanSApo). An excitation 636.5-nm laser (operated in continuous wave mode) was adjusted to ~40 μW to prevent photobleaching and decrease the formation of Atto655 triplet state (*20*). Emitted fluorescence signal was focused through a pinhole, split 50/50 by a beam splitter, passed through a 690/70-nm band-pass filter and detected by two single-photon avalanche photodiodes (SPADs). Cross-correlation of fluorescence trace was used to eliminate SPAD after-pulsing effects.

To ensure single-molecule detection within the confocal detection volume (~1 femtoliter), purified RNCs were diluted to ~10 nM in HiFi buffer. Each measurement used 50 μl of the RNC sample (~10 nM). Where indicated, TF (3 μM) was added to the RNC sample. All experiments were conducted at least two to three times with RNCs from independent preparations. For each RNC solution, four replicate measurements were recorded at consecutive 10-min intervals at room temperature (22°C). Fluorescence traces were analyzed by the SymPhoTime 64 software (PicoQuant). The ACF was calculated using 314 time points between 0 and 1 s. The reliability of each measurement was confirmed by comparing replicates, with overlapping diffusion curves indicating RNC stability during measurement. As the lateral dimension of the detection volume is much larger than the axial dimension, a two-dimensional single-species diffusion model was used to fit the ACF

$$G\left(\tau \right)=\left(1+c_{1}e^{-k_{1}t}+c_{2}e^{-k_{2}t}\right)\left(\frac{1-F+Fe^{-k_{f}t}}{1-F}\right)\left(\frac{1}{N}\right)\left(\frac{1}{1+k_{d}t}\right)$$

where *k*_1_ and *k*_2_ represent relaxation rate constants and *c*_1_ and *c*_2_ represent their respective amplitudes. *k_f_* denotes the rate constant of triplet state decay, with *F* as the corresponding amplitude. *k_d_* is the diffusion coefficient, and *N* represents the average number of molecules within the focal volume. Across all constructs, RNC diffusion and triplet state decay rates are similar, with relaxation times of ~1 ms and ~40 μs, respectively. The fitted parameter *N* was used to normalize the ACF to *N* = 1. After refitting the ACF, the *Y*-intercept *G*(0) was used to quantify the proportion of fluorescent molecules

$$G\left(0\right)=\left(1+c_{1}+c_{2}\right)\left(\frac{1}{1-F}\right)\left(\frac{1}{N}\right)$$

The DNC was calculated as

$$\text{DNC}=G{\left(0\right)}_{W6}-G{\left(0\right)}_{W6F}$$

The resulting dimensionless value provides a qualitative estimate for the fraction of dynamic nascent chains. The effect of mutations was estimated as

DNC change=DNC(mutant)−DNC(WT)

A detailed analysis of the fluctuation dynamics from the rate constants of the ACF was not performed, because the exact values of the calculated elemental rate constants are somewhat model dependent and, therefore, do not provide additional insights beyond the qualitative comparison of WT and mutants based on the *G*(0) values.

#### Limited proteolysis

All measurements were performed using RNCs labeled with N-terminal Bodipy FL. Proteolysis was initiated by mixing RNCs (0.02 μM) and thermolysin (0.5 ng/μl, Promega) in HiFi buffer supplemented with CaCl_2_ (0.25 mM). Reactions were incubated for the indicated times at 37°C and quenched by adding 1.75 volumes of quenching solution containing 85 mM tris-HCl (pH 6.8), 6% SDS, 1.7% 2-mercaptoethanol, 17% glycerol, and 0.3 M NaOH, followed by incubation at 85°C for 15 min (*9*). To prevent fluorophore bleaching, pH was adjusted to neutral by adding 2 M Hepes. Translation products were analyzed by tris-tricine SDS-PAGE and visualized via Bodipy fluorescence using the Amersham Typhoon RGB scanner (Cytiva). Band intensities on SDS-PAGE were quantified using the Multi Gauge software, and digestion time courses were evaluated by single-exponential fitting using GraphPad Prism (version 9.0.0).

#### Data analysis of the FPA

FPA mRNA constructs contained the N-terminal HemK codon sequence of varying length in one or two-codon increments, followed by 17 codons of the SecM arrest peptide and 20 codons of protein CspA (table S1). Under low-force conditions, translation is arrested at the SecM sequence, resulting in the formation of an arrested translation product (AR) (*20*). However, if nascent peptide folding generates force, then translation arrest is alleviated, leading to the production of a longer full-length peptide (FL) (fig. S6). Translation products were separated by SDS-PAGE, and the fraction of the FL product was calculated as

$$f_{\mathrm{FL}}=\frac{I_{\mathrm{FL}}}{I_{\mathrm{FL}}+I_{\mathrm{AR}}}$$

where *I*_FL_ and *I*_AR_ represent the band intensity of the respective products. Experiments were repeated three times, and means ± SD values were calculated.

#### Stopped-flow experiments

To monitor PET between the N-terminal Bodipy-FL and Trp residues in the polypeptide chain during real-time translation, stopped-flow PET experiments were performed at 37°C using an SX-20MV device (Applied Photophysics). Excitations was set to 470 nm (slits width of 2 mm, bandpass of 4.65 nm/mm), and emission was detected through a KV500 cutoff filter (Schott). Equal volumes of purified IC and freshly prepared TC were rapidly mixed to initiate the translation reaction, and fluorescence changes were recorded with 4000 time points over 2 min on a logarithmic scale. Fluorescence was normalized to the initial fluorescence, calculated from the averaged signal between 0.001 and 0.005 s. For each experiment, at least two biological replicates with six technical replicates each were averaged. PET efficiency was calculated according to the equation: *E*_PET_ = 1 − *F*_W6_/*F*_W6F_, where *F*_W6_ and *F*_W6F_ are fluorescence of the W6 and W6F constructs, respectively.

### Use of artificial intelligence

We used ChatGPT4.0 to improve the readability of the manuscript. The prompt we used was “edit grammar.” After using ChatGPT4.0, we reviewed and edited the content as needed and take full responsibility for the content of the manuscript.
