# Supplementary material for: Cotranslational protein folding through non-native structural intermediates
Source: Sci Adv. 2025 Sep 5;11(36):eady2211. doi: 10.1126/sciadv.ady2211 (PMC12412654; doi:10.1126/sciadv.ady2211)
Supplement: Supplementary file 1 — Figs. S1 to S6 Tables S1 and S2 [file sciadv.ady2211_sm.pdf]

Science Advances
AAAS

Supplementary Materials for

Cotranslational protein folding through non-native structural intermediates

Siyu Wang *et al.*

Corresponding author: Marina V. Rodnina, rodnina@mpinat.mpg.de;
Eugene I. Shakhnovich, shakhnovich@chemistry.harvard.edu

Sci. Adv. **11**, eady2211 (2025)
DOI: 10.1126/sciadv.ady2211

This PDF file includes:

Figs. S1 to S6
Tables S1 and S2

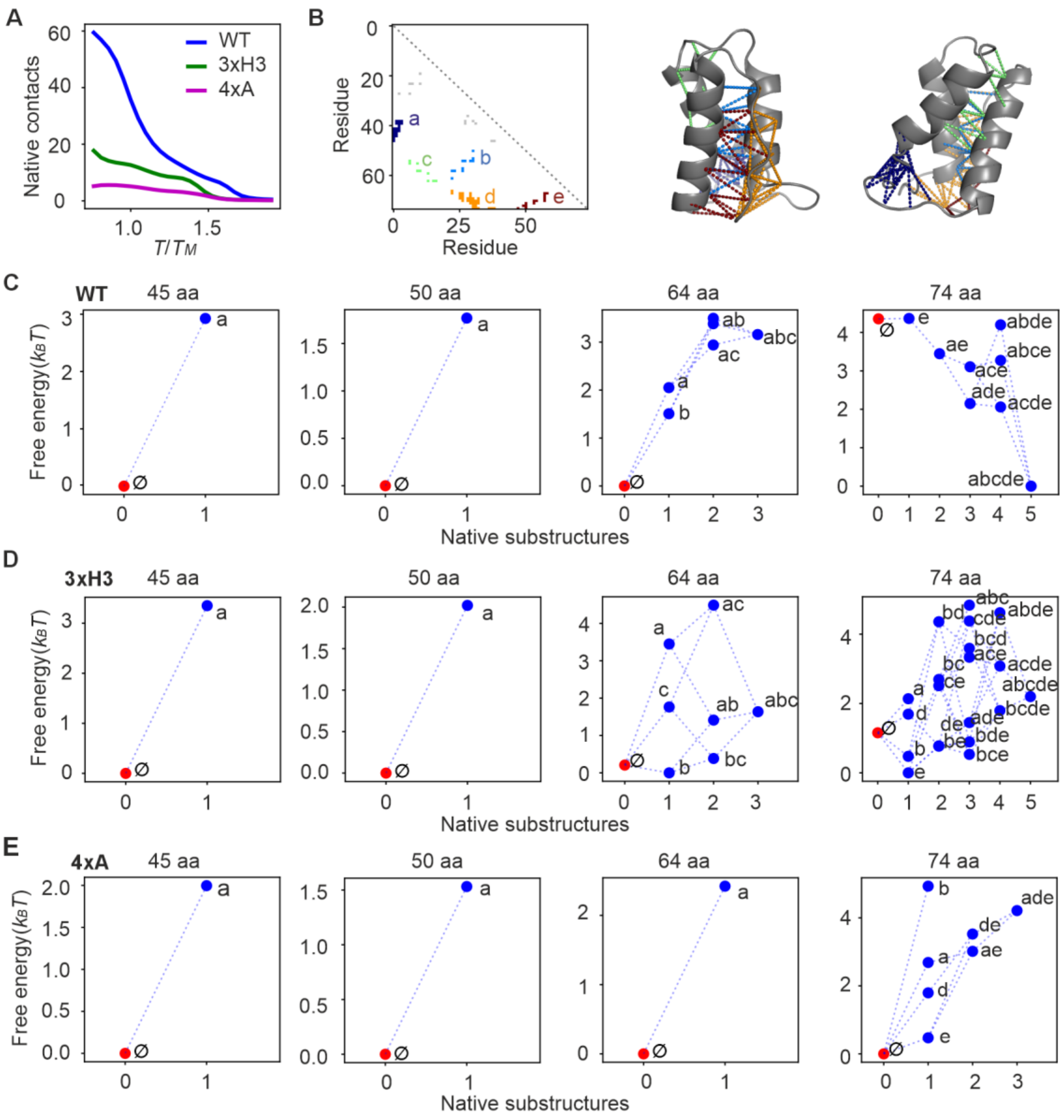

fig. S1. Simulation of equilibrium free energy landscapes during synthesis reveals the transition from non-native to native-like structure.

(**A**) Simulated thermal melting curves showing the equilibrium fraction of native contacts as a function of temperature for full-length (74 aa) WT HemK NTD and the 3xH3 and 4xA mutants. Temperature is normalized to the experimentally determined melting temperature (T_m) of WT HemK NTD (323 K). Simulation results are presented at T = 0.86 T_m, corresponding to in vitro experimental conditions at 293 K.

(**B**) Contact map of full-length HemK (74 aa), with folding substructures identified as groups of adjacent native contacts that cooperatively form and break. Substructures are color-coded and labeled alphabetically. Gray-shaded points indicate isolated contacts that do not belong to any substructure and are expected to form and break non-cooperatively. To the right, two views of the equilibrated HemK structure are shown, with contacts corresponding to substructures marked as colored dashes matching the contact map.

(**C**) Free energy landscapes of WT HemK as a function of topological configuration, defined by the subset of formed substructures, at different protein lengths. Each dot represents a single configuration, positioned on the X-axis by the number of substructures formed and on the Y-axis by its free energy (in $k_B T$). Red dots indicate configurations with no native structures (∅). Dots are connected if they differ by a single substructure, representing potential folding pathways. Notably, these configurations represent only native folding; for example, a snapshot containing a substantial number of non-native contacts is classified within a configuration with few or no substructures (∅ or a). At intermediate lengths (left panels), the landscape is dominated by non-native states, whereas at full length (right panel), native folding becomes thermodynamically favorable.
(**D, E**) Free energy landscapes for the 3xH3 (D) and 4xA (E) mutants. Native-like folding is unfavorable at all lengths. However, in the 3xH3 mutant, native-like intermediates (e.g., bce) are more favorable at full length compared to the 4xA mutant. In the 4xA mutant, the free energy difference between the native state (abcde) and the most stable non-native state (∅) exceeds the 5 $k_B T$ cutoff and is therefore not shown. Although 3xH3 residues are solvent-exposed in the native structure, they destabilize the full-length native state (D). In our simulations, local structural fluctuations transiently bring these hydrophobic residues into the core, stabilizing the native state in WT, whereas mutations disrupt this effect.

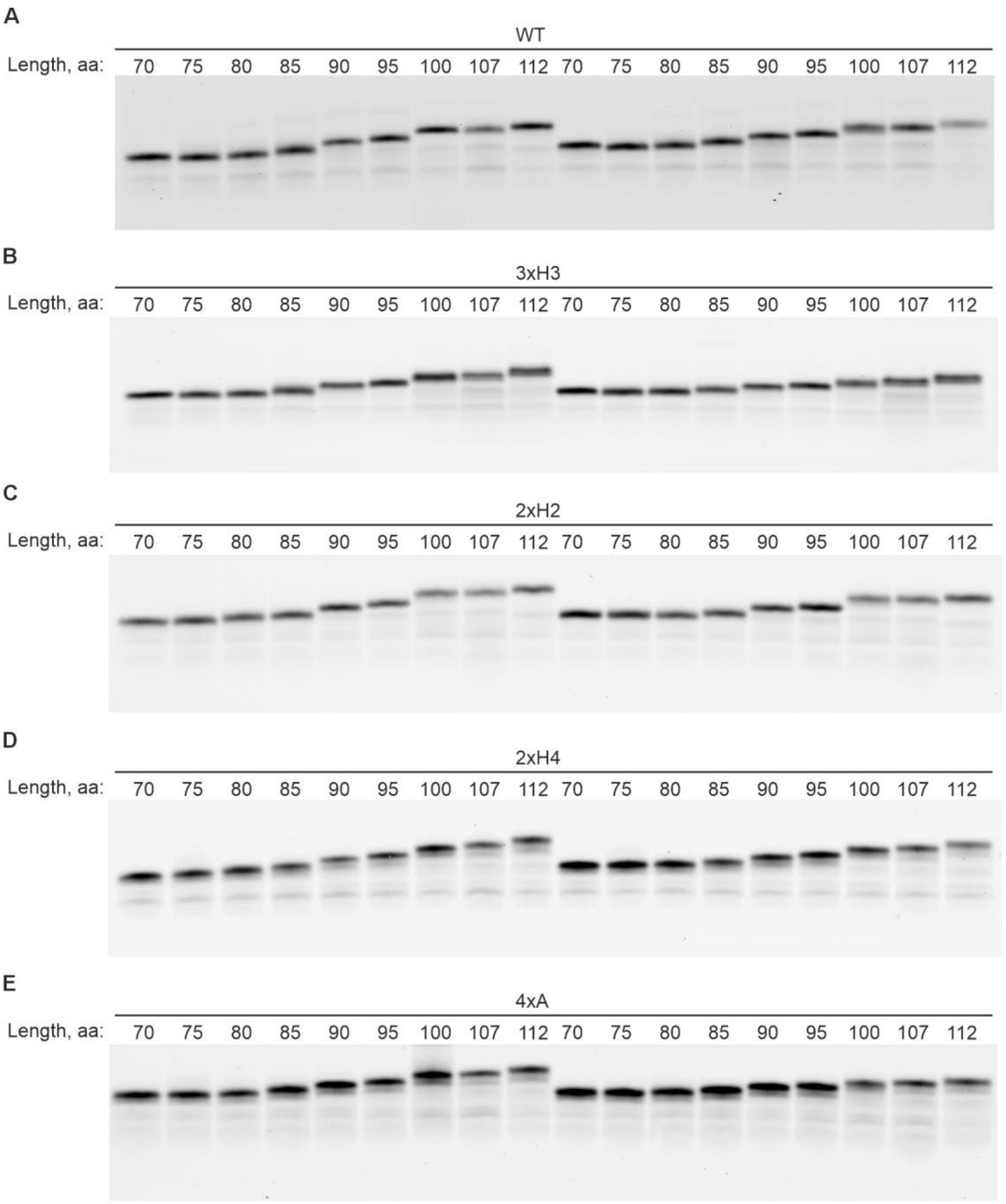

fig. S2. *In vitro* translation efficiency of WT, 3xH3, 2xH2, 2xH4, and 4xA mRNA constructs of different lengths.

Translation products were separated using Tris-Tricine SDS-PAGE and visualized via the fluorescence of the BODIPY-FL reporter attached to the N-terminus of the peptide. The aa lengths of the HemK constructs are indicated. (**A**) WT W6 and WT W6F. (**B**) 3xH3 W6 and 3xH3 W6F. (**C**) 2xH2 W6 and 2xH2 W6F. (**D**) 2xH4 W6 and 2xH4 W6F. (**E**) 4xA W6 and 4xA W6F.

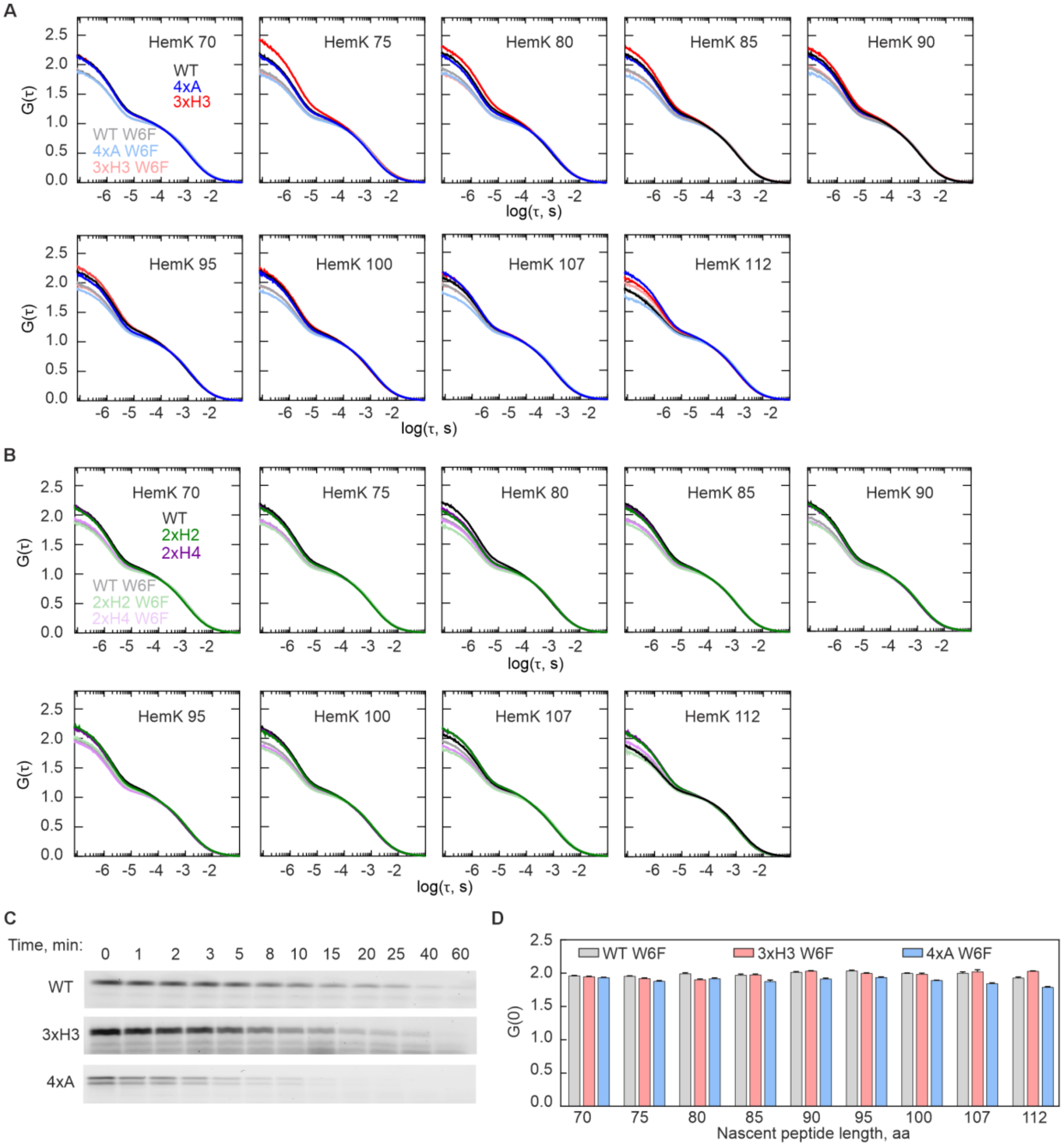

fig. S3. PET-FCS autocorrelation and proteolysis.

(**A, B**) PET-FCS autocorrelation function (ACF) curves for WT, 3xH3, and 4xA (**A**), as well as 2xH2 and 2xH4 (**B**), nascent chains at the indicated chain lengths. ACFs are averages of at least 8 biological replicates (N≥8). (**C**) Time courses of thermolysin proteolysis for WT, 3xH3, and 4xA 112-aa nascent chains, highlighting differences in protease sensitivity. (**D**) G(0) values of ACFs for W6F constructs of WT (grey), 3xH3 (pink) and 4xA(blue) reflecting the extent of intermolecular PET quenching of the N-terminal BodipyFL by the ribosome. Error bars represent SEM calculated from at least 8 biological replicates (N⩾8).

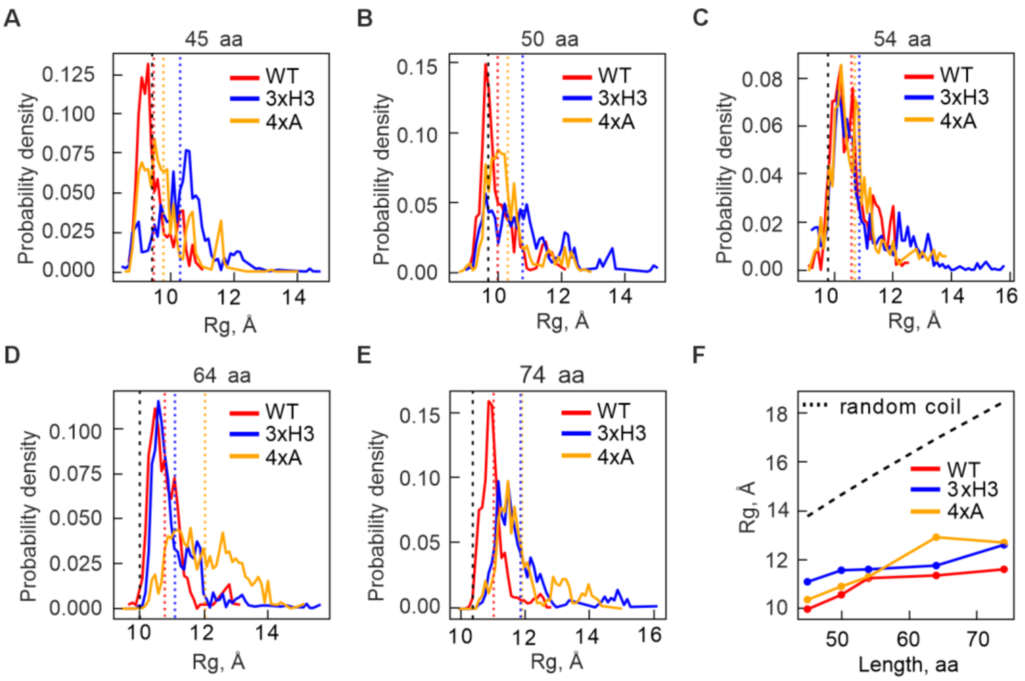

fig. S4. Non-native interactions maintain compactness of early folding intermediates.

(**A-E**) Probability density plots of the radius of gyration (R_g) as a function of peptide length for WT HemK (red), 3xH3 mutant (blue), and 4xA mutant (orange). Vertical dashed lines indicate the average R_g for each construct in the corresponding color, while the expected R_g for native-like folding at each length is shown as a vertical dashed black line. Peaks in the distributions suggest the presence of multiple interconverting states with distinct average R_g values, leading to non-symmetric R_g distributions rather than the symmetric distributions expected for a single folding state.

(**F**) Average R_g as a function of chain length for WT, 3xH3, and 4xA mutants, as well as expected value for a random coil control, illustrating differences in compactness due to non-native interactions in the mutants.

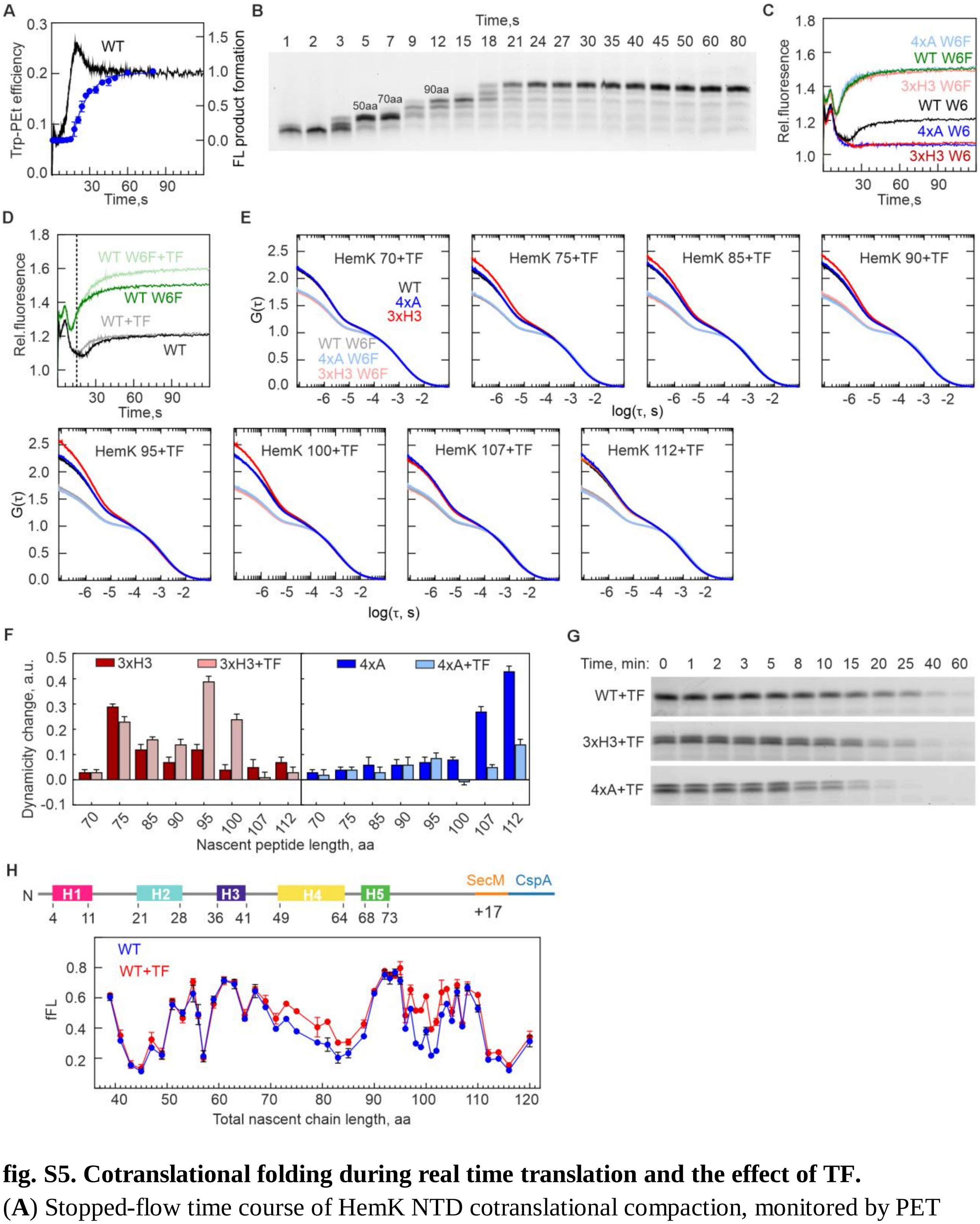

fig. S5. Cotranslational folding during real time translation and the effect of TF.

(**A**) Stopped-flow time course of HemK NTD cotranslational compaction, monitored by PET between BodipyFL-Met1 and Trp6 in the nascent chain (black), together with the time course of

full length product formation (blue). Error bars time course trace are SEM calculated from 3 biological replicates (N=3).

(**B**) Translation time course for WT mRNA. The lengths of transiently-accumulating translation are indicated. (**C**) Relative fluorescence changes of N-terminal BodipyFL emission during translation, showing intermolecular PET measured with the W6F variants compared to combined inter- and intramolecular PET observed with the W6 variants across different mutants. (**D**) Changes in BOF-Met fluorescence during translation of WT mRNA (black) and W6F mRNA (green) in the absence of TF, and in the presence of TF (gray and light green, respectively). The dashed line marks the time point of TF's initial interaction with the nascent peptide. (**E**) PET-FCS ACF curves at the indicated nascent peptide length in the presence of TF. ACFs are averages from at least 8 biological replicates (N≥8). (**F**) DNC changes relative to WT for 3xH3 (red) and 4xA (blue) mutants in the absence and presence of TF. Error bars represent SEM calculated from at least 8 biological replicates (N⩾8).(**G**) Time courses of thermolysin proteolysis for WT, 3xH3, and 4xA 112-aa nascent chains in the presence of TF, highlighting differences in protease sensitivity. (**H**) FPA profile of the WT nascent peptide in the presence (red) and absence (blue) of TF. The total nascent chain length includes both the HemK nascent peptide and the SecM stalling sequence. Data represent the mean ± SD from three biological replicates (N=3). A schematic of the FPA experiment construct is shown on top (see Methods for details).

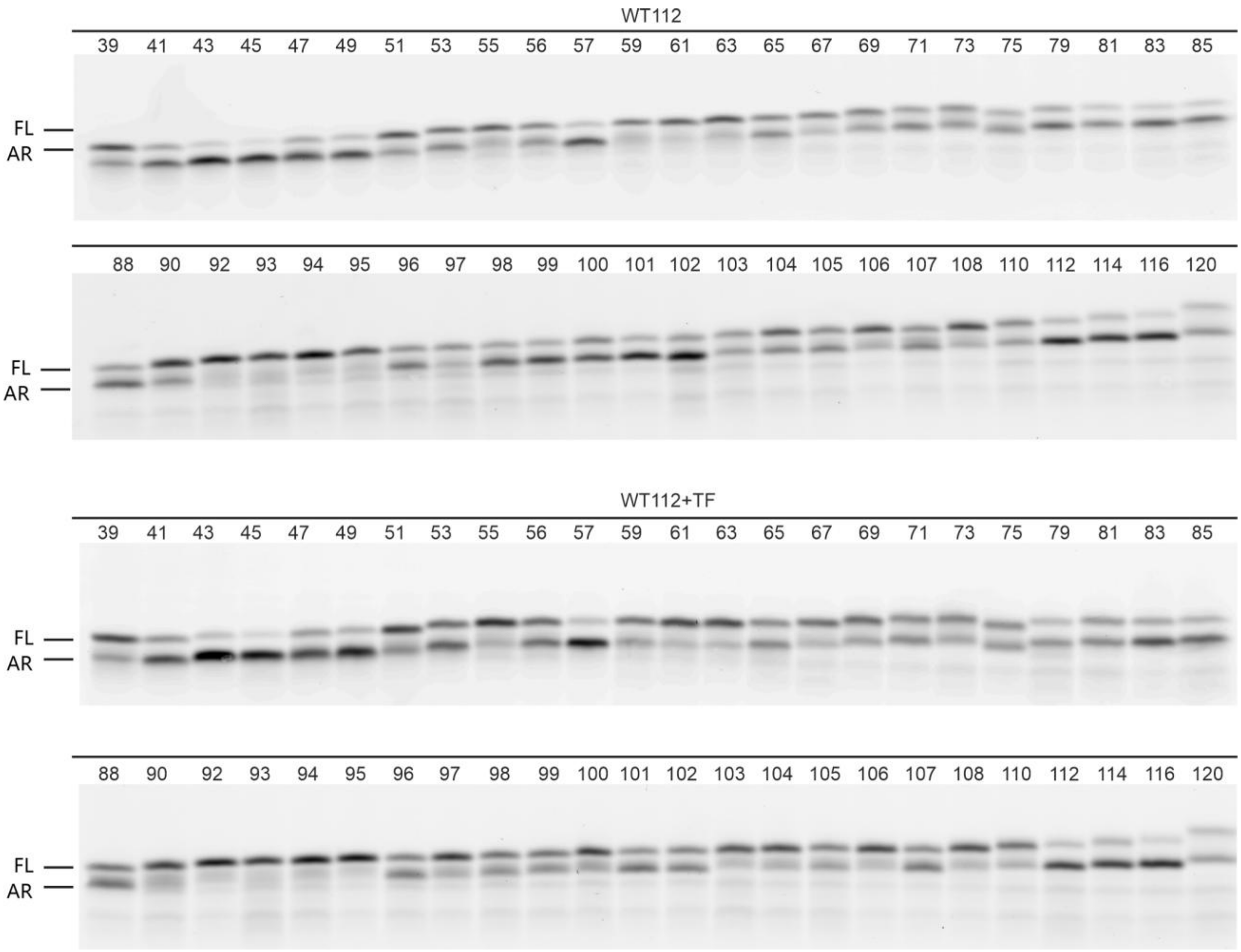

fig. S6. FPA analysis. SDS-PAGE of translation products shows two bands which were used to calculate the fraction of full length (FL) compared to total FL and arrest (AR) peptide in Fig 4F.

table S1. List of aa sequences of protein constructs studied by PET-FCS and FPA .

HemK	Full length 112-aa constructs
WT	MEFQHWLREA ISQLQASESP RRDAEILLEH VTGKGRTFIL<br>AFGETQLTDE QCQQLDALLT RRRDGEPIAH LTGVREFFSL<br>PLFVSPATLI PRPDTECLVE QALARLPEQP CR
WT W6F	MEFQHFLREA ISQLQASESP RRDAEILLEH VTGKGRTFIL<br>AFGETQLTDE QCQQLDALLT RRRDGEPIAH LTGVREFFSL<br>PLFVSPATLI PRPDTECLVE QALARLPEQP CR
3xH3 (F38A_L40A_F42A)	MEFQHWLREA ISQLQASESP RRDAEILLEH VTGKGRTAIA<br>AAGETQLTDE QCQQLDALLT RRRDGEPIAH LTGVREFFSL<br>PLFVSPATLI PRPDTECLVE QALARLPEQP CR
3xH3 W6F<br>(F38A_L40A_F42A)	MEFQHFLREA ISQLQASESP RRDAEILLEH VTGKGRTAIA<br>AAGETQLTDE QCQQLDALLT RRRDGEPIAH LTGVREFFSL<br>PLFVSPATLI PRPDTECLVE QALARLPEQP CR
2xH2<br>(L27A_L28A)	MEFQHWLREA ISQLQASESP RRDAEIAAEH VTGKGRTFIL<br>AFGETQLTDE QCQQLDALLT RRRDGEPIAH LTGVREFFSL<br>PLFVSPATLI PRPDTECLVE QALARLPEQP CR
2xH2 W6F<br>(L27A_L28A)	MEFQHFLREA ISQLQASESP RRDAEIAAEH VTGKGRTFIL<br>AFGETQLTDE QCQQLDALLT RRRDGEPIAH LTGVREFFSL<br>PLFVSPATLI PRPDTECLVE QALARLPEQP CR
2xH4<br>(L55A_L58A)	MEFQHWLREA ISQLQASESP RRDAEILLEH VTGKGRTFIL<br>AFGETQLTDE QCQQADAALT RRRDGEPIAH LTGVREFFSL<br>PLFVSPATLI PRPDTECLVE QALARLPEQP CR
2xH4 W6F<br>(L55A_L58A)	MEFQHFLREA ISQLQASESP RRDAEILLEH VTGKGRTFIL<br>AFGETQLTDE QCQQADAALT RRRDGEPIAH LTGVREFFSL<br>PLFVSPATLI PRPDTECLVE QALARLPEQP CR
4xA<br>(L27A_L28A_ L55A_L58A)	MEFQHWLREA ISQLQASESP RRDAEIAAEH VTGKGRTFIL<br>AFGETQLTDE QCQQADAALT RRRDGEPIAH LTGVREFFSL<br>PLFVSPATLI PRPDTECLVE QALARLPEQP CR
4xA W6F<br>(L27A_L28A_ L55A_L58A)	MEFQHFLREA ISQLQASESP RRDAEIAAEH VTGKGRTFIL<br>AFGETQLTDE QCQQADAALT RRRDGEPIAH LTGVREFFSL<br>PLFVSPATLI PRPDTECLVE QALARLPEQP CR
WT HemK (FPA)	MEYQHWLREA ISQLQASESP RR$_{22}$ DA$_{24}$ EI$_{26}$ LL$_{28}$EH$_{30}$VT$_{32}$GK$_{34}$<br>GR$_{36}$TF$_{38}$I$_{39}$L$_{40}$ AF$_{42}$GE$_{44}$TQ$_{46}$LT$_{48}$DE$_{50}$ QC$_{52}$QQ$_{54}$ LD$_{56}$AL$_{58}$LT<br>RR$_{62}$RD$_{64}$GE$_{66}$PI$_{68}$AHL$_{71}$TG$_{73}$VR$_{75}$E$_{76}$F$_{77}$W$_{78}$S$_{79}$L $_{80}$P$_{81}$L$_{82}$F$_{83}$V$_{84}$<br>S$_{85}$P$_{86}$A$_{87}$T$_{88}$L $_{89}$I $_{90}$P$_{91}$RP$_{93}$DT$_{95}$EC$_{97}$LV$_{99}$ EQ$_{101}$ FSTPVWIS<br>QAQGIRAGPMSGKMTGIVK WFNADKGFGFITP

Constructs with W6F replacements (*blue*) serve as controls for intermolecular quenching interactions with the ribosome. Point mutation sites are indicated in *red*. In all constructs, additional Y3F and W78F substitutions (underlined) were introduced to prevent PET. Constructs truncations in HemK are denoted by subscript numbers, followed by the SecM sequence (green) and CspA (purple).

table S2. Primers used to generate mRNA transcription templates

Primer	Primer sequence (5’-3’)
T7 forward primer	TAATACGACTCACTATA
HemK70 rev	ATGAGCAATGGGTTCACCATCGCGACGAC
HemK75 rev	TCGCACCCCGGTTAAATGAGCAATGGG
HemK80 rev	CAACGAAAAGAATTCTCGCACCCCGG
HemK85 rev	CGAAACAAATAACGGCAACGAAAAGAATTC
HemK90 rev	AATTAAGGTCGCTGGCGAAACAAATAAC
HemK95 rev	CGTATCCGGGCGCGGAATTAAGGTCGC
HemK100 rev	CTCCACCAGACACTCCGTATCCGGGCG
HemK107 rev	AGGCAACCGCGCCAGTGCCTGCTCCACC
HemK112 rev	ACGGCAAGGTTGTTCAGGCAACCGCGCC
CspA20 mRNA rev	AGGAGTGATGAAGCCGAAGCCT
